# Prognostic Significance of Tumour Budding and Desmoplastic Reaction in Intestinal-Type Gastric Adenocarcinoma

**DOI:** 10.1177/10668969221105617

**Published:** 2022-06-20

**Authors:** Cherry Pun, Shelly Luu, Carol Swallow, Richard Kirsch, James R. Conner

**Affiliations:** 1 Department of Laboratory Medicine and Pathobiology, 7938 University of Toronto, Toronto, Ontario, Canada; 2 Department of Surgery, University of Toronto, Lunenfeld-Tanenbaum Research Institute, 518775 Sinai Health System, Toronto, Ontario, Canada; 3 Department of Pathology and Laboratory Medicine, 518775 Sinai Health System, Toronto, Ontario, Canada

**Keywords:** tumour budding, desmoplastic reaction, stromal maturity, gastric adenocarcinoma

## Abstract

*Objective*. Tumour budding and desmoplastic reactions in peritumoural stroma are features of the tumour microenvironment that are associated with colorectal cancer prognosis but have not been as thoroughly examined in gastric cancer. We aimed to further characterize the prognostic role of tumour budding and desmoplastic reaction in gastric adenocarcinoma with intestinal differentiation. *Methods*. 76 curative gastrectomy specimens were identified, excluding post-neoadjuvant cases or cases with >50% diffuse-type histology. Tumour budding was defined and graded according to the International Tumor Budding Consensus Conference recommendations and desmoplastic reaction was classified as described by Ueno et al 2017. Tumour budding and desmoplastic reaction were analyzed for associations with pathologic features and clinical outcomes. *Results*. Tumour budding was associated with pT (*P* < .001), pN (*P* < .004), overall stage (*P* < .001), LVI (*P* < .001) and PNI (*P* = .002). Desmoplastic reaction was associated with pT (*P* < .001), pN (*P* = .005), overall stage (*P* = .031) and PNI (*P* < .001), but not LVI. Survival analysis showed decreased overall survival (OS) and recurrence-free survival (RFS) for intermediate and high grade tumour budding (*P* = .031, .014 respectively). Immature stroma was significantly associated with RFS but not OS. Neither tumour budding nor desmoplastic reaction were independent predictors of OS or RFS on multivariate analysis in this cohort. *Conclusion*. Tumour budding and desmoplastic reaction were associated with known pathologic risk factors. Prognostically, tumour budding was associated with OS and RFS while desmoplastic reaction was associated with RFS only. Our data suggest that tumour budding and desmoplastic reaction have prognostic value in intestinal-type gastric adenocarcinoma.

## Introduction

Gastric adenocarcinoma is the sixth most common cancer in the world and third leading cause of death.^1^ The TNM classification system (AJCC eighth edition), based on depth of invasion, nodal involvement, and distant metastasis is used in addition to other prognostic factors such as Lauren classification and lymphatic, vascular, and perineural invasion to estimate prognosis of gastric adenocarcinoma patients after resection.^2^ Despite such efforts to identify high-risk patients, survival outcomes remain heterogenous and even some early-stage patients may have poor outcomes. Additional prognostic markers would thus be useful to further stratify prognostic subgroups.

Tumour budding and desmoplastic reaction in peritumoural stroma are features of the tumour microenvironment that have been linked to prognosis in several cancer types, most prominently colorectal cancer.^3–12^ Tumour budding, defined as single cells or clusters up to four cells present at the invasive front of the tumour, is thought to be a histological representation of epithelial-mesenchymal transition, which is integral to the development of a promigratory phenotype important for invasion and metastasis.^13–16^ As such, tumour budding has been identified as an independent prognostic factor in malignant colorectal polyps and in stage II and III colorectal carcinoma. The International Tumor Budding Consensus Conference (ITBCC) established guidelines for assessing tumour budding in colorectal carcinoma, which has recently been incorporated into the College of American Pathologists’ Cancer Protocol Templates.^5^

Characteristics of desmoplastic reaction have also been shown to be associated with prognosis in colorectal carcinoma, although reporting of this feature is not currently addressed in most cancer protocols.^6–12^ Ueno and colleagues classified desmoplastic reaction into a three-tier system of mature, intermediate, and immature stroma, and found that intermediate and immature stroma were associated with poorer clinical outcomes in colorectal carcinoma patients compared to mature stroma.^6–8,12^

The significance of tumour budding and desmoplastic reaction has not been as thoroughly examined in gastric adenocarcinoma, though several studies have suggested that they may have prognostic significance similar to that in colorectal carcinoma.^17–27^ The scoring methods of tumour budding have varied across these studies, with two recent reports applying the ITBCC recommendations in their assessment of tumour budding.^26,27^ Furthermore, several of these studies include tumour budding assessment in diffuse-type gastric adenocarcinoma, which often exhibit high-grade tumour budding, and this may confound the prognostic power in patients with intestinal-type gastric adenocarcinoma.^22^ Currently, there is no consensus for the assessment of desmoplastic reaction in either colorectal carcinoma or gastric adenocarcinoma, though the three-tier system proposed by Ueno and colleagues has consistently shown independent prognostic value in stage II colorectal carcinoma across multiple cohorts.^12^ In this study we further characterized their prognostic role in a cohort limited to gastric adenocarcinoma with at least 50% intestinal differentiation using established ITBCC guidelines for tumour budding and the same criteria applied by Ueno and colleagues in colorectal carcinoma for desmoplastic reaction.

## Methods

### Case Selection and Data Collection

In this single-institution retrospective study, we identified 76 patients with intestinal-type gastric adenocarcinoma treated with primary gastrectomy between 2000 and 2018 at Mount Sinai Hospital, Toronto, Canada. Patients undergoing non-curative surgery (i.e. debulking only) and patients who received neoadjuvant therapy were excluded. Tumours classified by Lauren criteria as “mixed-type” were included for analysis if they had at least 50% intestinal differentiation. Patients were followed up to five years while patients with less than 30 days of follow-up were excluded in survival analysis to omit poor outcomes due to surgical complications. Clinicopathological data including sex, age at diagnosis, tumour depth of invasion (pT stage), number of nodal metastases (pN stage), overall TNM stage, lymphovascular (LVI), perineural invasion (PNI), overall survival (OS) and recurrence-free survival (RFS) outcomes were collected from the electronic patient record and pathology reports. Hematoxylin and eosin (H&E) stained slides were reviewed by one of the authors, a pathology resident with interest in gastrointestinal pathology (C.P.), to assess tumour budding and desmoplastic reaction, after a training set of 20 slides were reviewed with two practicing gastrointestinal pathologists (J.C. and R.K.) with consensus. This study was approved by the institutional Research Ethics Board.

### Tumour Budding

**Figure 1.**
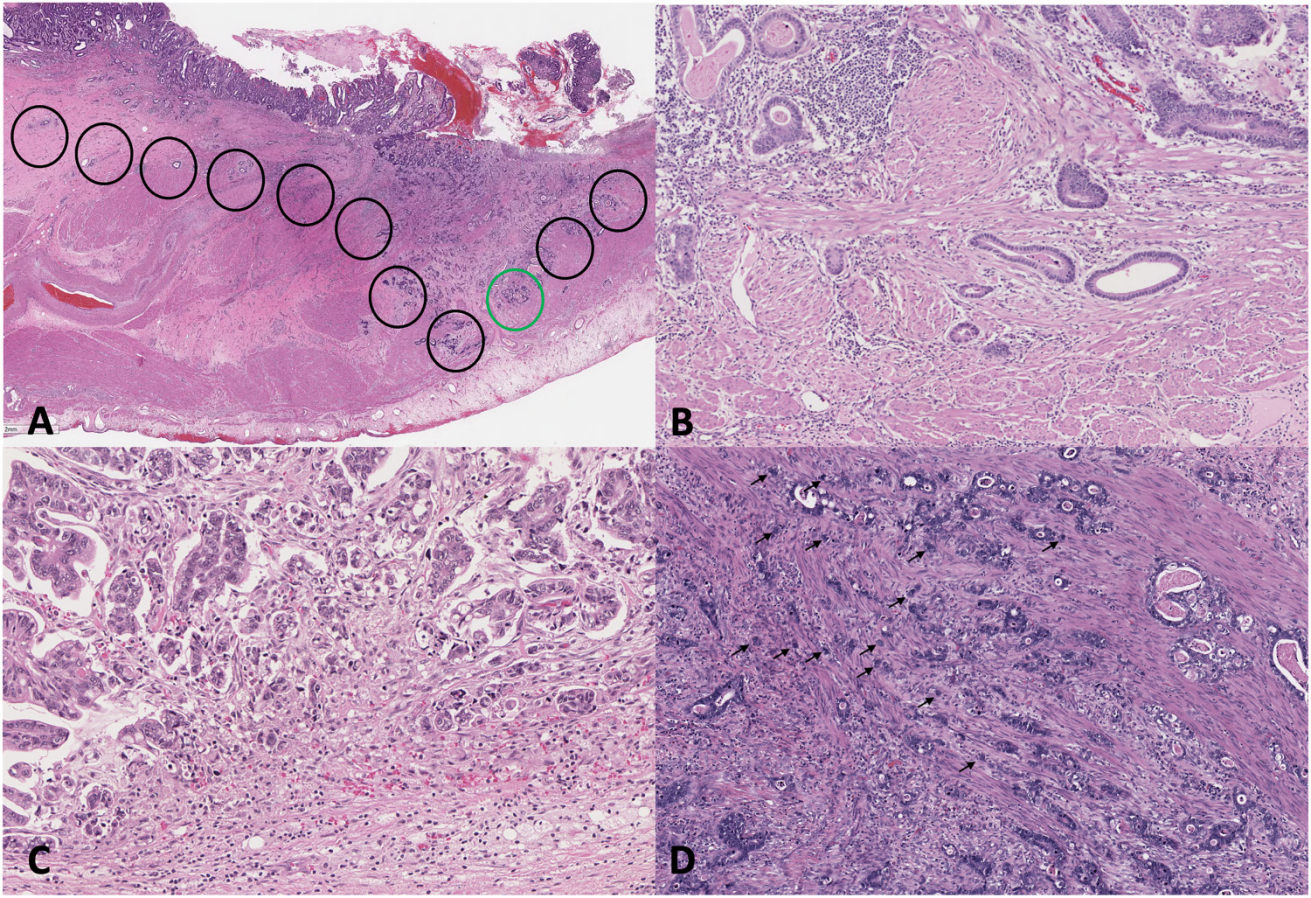
Tumour budding (tumour budding) in intestinal-type gastric adenocarcinoma. A. International Tumor Budding Consensus Conference (ITBCC) method of scanning tumour at the invasive front at x100 (black circles) for the hotspot containing the highest degree of tumour budding (green circle), which is then assessed at x200 to obtain the raw bud count. Tumour budding grade is then classified by applying the normalization factor into the following categories: B. low-grade budding (Bd1, 0-4 buds), C. intermediate-grade budding (Bd2, 5-9 buds), and D. high-grade budding (Bd3, ≥10 buds). Hematoxylin-eosin stains, A, x10, and B, C, and D, x200.

Tumour budding grade was defined based on the ITBCC 2016 recommendations for colorectal cancer and evaluated using the hot-spot method at the invasive front of the tumour (Figure 1 A-D).^5^ Tumour budding was assessed in a single 20x objective with a 22 mm field diameter and divided by the normalization factor (1.210 based on a 22 mm eyepiece field diameter) to obtain the standardized bud count. The standardized bud count was graded into three tiers: low-grade Bd1 (0-4 buds), intermediate-grade Bd2 (5-9 buds), and high-grade Bd3 ( ≥ 10 buds). Only H&E slides were used to assess tumour budding. In cases with significant peritumoural inflammation, which may obscure assessment of buds, the hot-spot slide was reviewed collaboratively by two pathologists (J.C. and R.K.) to achieve consensus of tumour budding grade. In cases with a minor component of diffuse-type histology, tumour budding was only assessed in the areas of intestinal-type differentiation.

### Peritumoural Stroma

**Figure 2.**
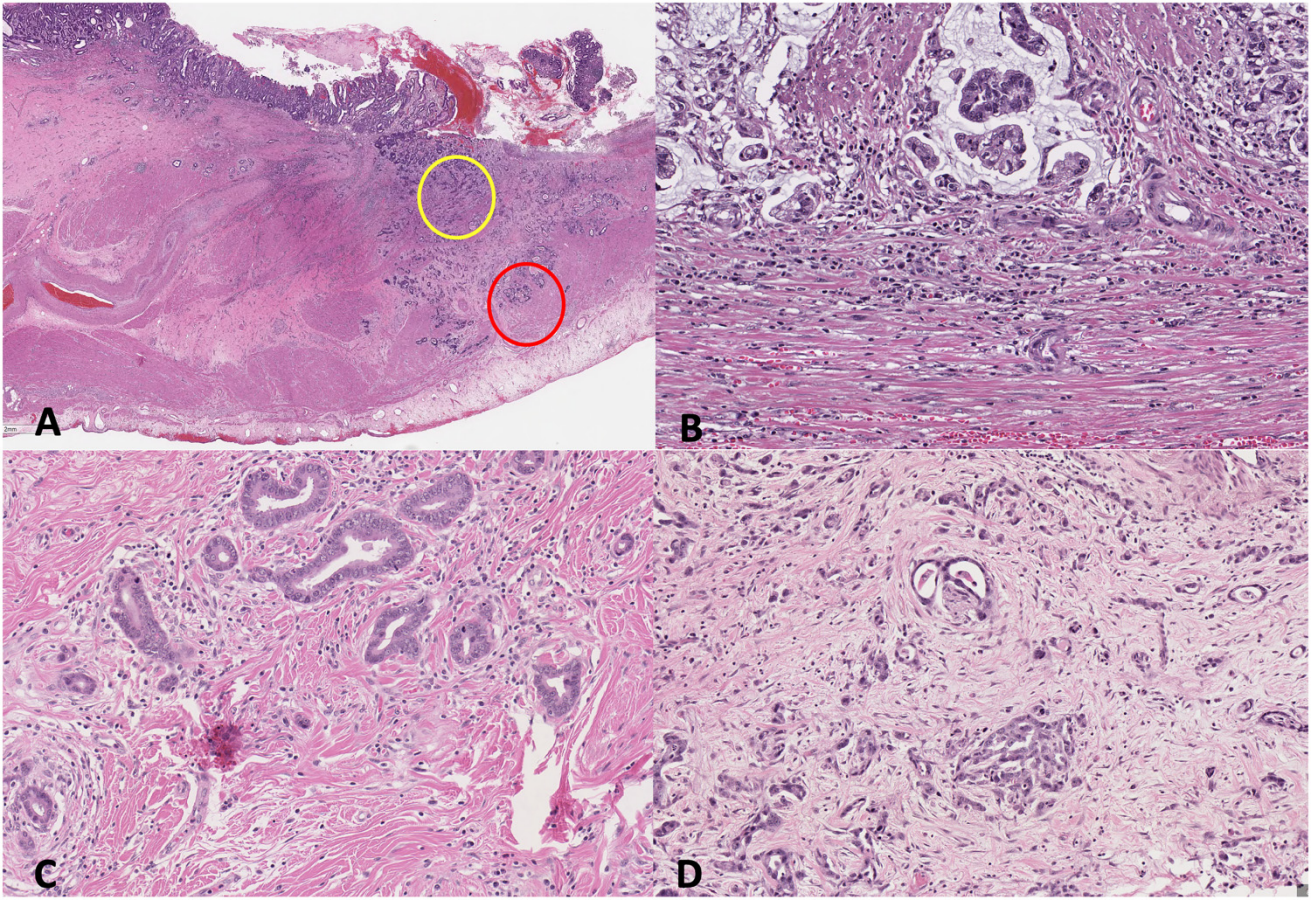
Desmoplastic reaction in intestinal-type gastric adenocarcinoma. A. desmoplastic reaction is assessed on low power at both the invasive front (red circle) or within the tumour (yellow circle) for areas of myxoid stroma or keloid-like collagen. B. Mature stroma comprised of densely packed fine collagen fibres. C. Intermediate stroma, consisting of areas of keloid-like collagen, fulfilling at least one x400 field. D. Immature stroma with myxoid changes, fulfilling at least one x400 field. Hematoxylin-eosin, A, x10, and B, C, and D, x200.

Desmoplastic reaction was evaluated as described by Ueno et al, 2017 for colorectal carcinoma, in which the stroma was evaluated at the invasive front of the tumour.^7^ In addition, stroma within the main tumour mass was also evaluated as demonstrated by Kemi et al, 2018.^23^ Cases were classified as mature (DR1; fine, densely packed collagen fibres), intermediate (DR2; keloid-like collagen), or immature (DR3; myxoid stroma), with intermediate or immature stroma needing to fill at least one 40x field to be classified as such (Figure 2 A-D). The desmoplastic reaction classification was determined by the least mature stroma type present. In early-stage, or intramucosal gastric adenocarcinoma (less than pT1b), desmoplastic reaction was not assessed as stromal changes would not be evident until the tumour invades into the submucosa, whereas tumour budding was assessed in all T stages.

### Analysis

Tumour budding and desmoplastic reaction grading and classification were evaluated for association with pathologic prognostic factors, OS and RFS. Data were analyzed using IBM SPSS Statistics Version 27. Chi-squared test of independence (for ordinal variables) and one-way ANOVA (for non-ordinal variables) were used to analyze associations of clinicopathological variables between tumour budding grades and desmoplastic reaction classification. Survival analysis was performed using the Kaplan-Meier method (log rank test) and Cox Regression for uni- and multivariate analyses. A p-value of 0.05 was used for determining statistical significance.

## Results

### Clinicopathological Characteristics

The study group was comprised of 76 surgically treated patients with gastric adenocarcinoma who did not undergo neoadjuvant therapy (Table 1). The median and mean ages of diagnosis for all patients were 74 and 72.5 years, respectively (range: 50 to 92 years). The cohort consisted of 71% males (n = 54) and 29% females (n = 22). The mean postoperative follow-up time was 41 months (range: 5 to 60 months). Only 14 patients had HER2 testing performed, and 4 patients had a positive result while the remaining 10 were negative. Most patients were pathologic stage pT3 (63%), followed by pT4 (21%), pT1 (19%) and pT2 (16%) and pN0 (36%), followed by pN2 (28%), pN1 (25%) and pN3 (11%) (Table 1). At the time of gastrectomy, there were 4 patients who had M1 disease (5%) who underwent resections of additional areas with curative intent, including pancreas, diaphragm, small bowel, and mesentery. Overall, 50 (66%) cases showed lymphovascular invasion and 34 (45%) cases showed perineural invasion.

**Table 1.** Clinicopathological Characteristics and Association with TB and DR.

		n	(%)	Bd1 n = 16 (%)	Bd2 n = 16 (%)	Bd3 n = 44 (%)	P-value	DR1 n = 18 (%)	DR2 n = 20 (%)	DR3 n = 34 (%)	P-value
Age (median)	(74)			73.0	71.2	72.7	*0.795^a^*	71.5	71.5	73.0	*0.662^a^*
Sex	F	22	29%	4 (25%)	4 (25%)	14 (32%)	*0.811^b^*	6 (30%)	5 (25%)	10 (30%)	*0.852^b^*
	M	54	71%	12 (75%)	12 (75%)	30 (68%)		14 (70%)	15 (75%)	24 (70%)	
pT stage	T1A	4	5%	4 (25%)	0	0	***<0.001*** ***^b^***	0	0	0	***0.001*** ***^b^***
	T1B	11	14%	6 (38%)	3 (19%)	2 (5%)		8 (44%)	2 (10%)	1 (3%)	
	T2	12	16%	2 (13%)	5 (31%)	5 (11%)		3 (17%)	6 (30%)	3 (9%)	
	T3	33	43%	3 (19%)	4 (25%)	26 (60%)		5 (28%)	10 (50%)	18 (53%)	
	T4A	11	14%	1 (6%)	2 (13%)	8 (18%)		2 (11%)	0	9 (26%)	
	T4B	5	7%	0	2 (13%)	3 (7%)		0	2 (10%)	3 (9%)	
pN stage	N0	28	37%	11 (69%)	9 (56%)	8 (18%)	***0.004*** ***^b^***	9 (50%)	10 (50%)	5 (15%)	***0.005*** ***^b^***
	N1	19	25%	3 (19%)	4 (25%)	12 (27%)		7 (39%)	5 (25%)	7 (21%)	
	N2	21	28%	2 (13%)	3 (19%)	16 (36%)		2 (11%)	3 (15%)	16 (47%)	
	N3	8	11%	0	0	8 (18%)		0	0	6 (18%)	
	N-	28	37%	11 (69%)	9 (56%)	8 (18%)	***<0.001*** ***^b^***	9 (50%)	10 (50%)	5 (15%)	***0.007*** ***^b^***
	N +	48	63%	5 (31%)	7 (44%)	36 (82%)		9 (50%)	10 (50%)	29 (85%)	
M stage	M0	14	18%	2 (13%)	4 (25%)	8 (18%)	*0.802^b^*	3 (17%)	5 (25%)	6 (18%)	*0.441^b^*
	M1	4	8%	1 (6%)	1 (6%)	2 (5%)		0	3 (15%)	3 (9%)	
	Mx	56	74%	13 (81%)	10 (62%)	33 (75%)		15 (83%)	12 (60%)	25 (74%)	
TNM Stage	IA	10	13%	8 (50%)	1 (6%)	1 (2%)	***<0.001*** ***^b^***	4 (22%)	2 (10%)	0	***0.031*** ***^b^***
	IB	11	14%	2 (13%)	7 (44%)	2 (5%)		5 (28%)	5 (25%)	1 (3%)	
	IIA	15	20%	5 (31%)	2 (13%)	8 (18%)		5 (28%)	3 (15%)	7 (21%)	
	IIB	11	14%	0	2 (13%)	9 (20%)		2 (11%)	4 (20%)	5 (15%)	
	IIIA	13	17%	1 (6%)	1 (6%)	11 (25%)		2 (11%)	2 (10%)	9 (26%)	
	IIIB	9	12%	0	1 (6%)	8 (18%)		0	2 (10%)	7 (21%)	
	IIIC	2	3%	0	0	2 (5%)		0	0	2 (6%)	
	IV	5	7%	0	2 (13%)	3 (7%)		0	2 (10%)	3 (9%)	
LVI	N	26	34%	12 (75%)	7 (44%)	7 (16%)	***<0.001*** ***^b^***	6 (33%)	9 (45%)	7 (21%)	*0.163^b^*
	Y	50	66%	4 (25%)	9 (56%)	37 (84%)		12 (67%)	11 (55%)	27 (80%)	
PNI	N	42	55%	14 (88%)	11 (69%)	17 (39%)	***0.002*** ***^b^***	15 (83%)	14 (70%)	9 (26%)	***<0.001*** *^b^*
	Y	34	45%	2 (13%)	5 (31%)	27 (61%)		3 (17%)	6 (30%)	25 (74%)	
Died	N	43	57%	14 (88%)	9 (56%)	20 (45%)	***0.015*** ***^b^***	13 (72%)	10 (50%)	16 (47%)	*0.202^b^*
	Y	33	43%	2 (13%)	7 (44%)	24 (55%)		5 (28%)	10 (50%)	18 (53%)	
Recurred	N	48	63%	15 (94%)	11 (69%)	22 (50%)	***0.007*** ***^b^***	15 (83%)	14 (70%)	15 (44%)	***0.014*** ***^b^***
	Y	28	37%	1 (6%)	5 (31%)	22 (50%)		3 (17%)	6 (30%)	19 (56%)	

Abbreviations: TB, tumour budding; DR, desmoplastic reaction grade; Bd, tumour budding grade; LVI, lympho-vascular invasion; PNI, perineural invasion.

^a^ One-way ANOVA. ^b^Chi^2^ test of independence.

### Tumour Budding

Assessment of tumour budding using the ITBCC method revealed 44 patients (58%) with Bd3, 16 patients (21%) with Bd2, and 16 patients (21%) with Bd1 (Table 1). High-grade budding (Bd3) was associated with a higher pT stage (*P* < .001), pN stage (*P* *=* .004), overall TNM stage (*P* < .001), presence of LVI (*P* < .001) and PNI (*P* *=* .002) as shown in Table 1. Eleven patients had tumours classified by Lauren criteria as “mixed-type,” with areas containing both intestinal and diffuse-type components (as noted in the Methods section, only tumors from this group with at least 50% intestinal differentiation were included). Nine of these eleven patients (82%) had high-grade budding within the intestinal component, one patient had Bd2, and one had Bd1.

### Desmoplastic Reaction

Desmoplastic reaction was assessed in 72 patients (after excluding 4 pT1a cases): 18 patients (25%) showed mature stroma, 20 patients (28%) showed intermediate stroma, and 34 patients (47%) showed immature stroma (Table 1). Intermediate and immature stroma was present at the invasive front of the tumour in 50 patients while 22 patients had these features present within the main tumour mass. Immature stroma present in either location was associated with a higher pT stage (*P* = .001), pN stage (*P* = .005), overall TNM stage (*P* < .031), and PNI (*P* < .001), but was not significantly associated with lymphovascular invasion (*P* *=* .163, Table 1). Immature stroma was also associated with higher tumour budding grade (*P* < .001), in keeping with the model that both tumour budding and desmoplastic reaction represent characteristics of the tumour microenvironment associated with prognosis.

### Survival Analysis

**Figure 3.**
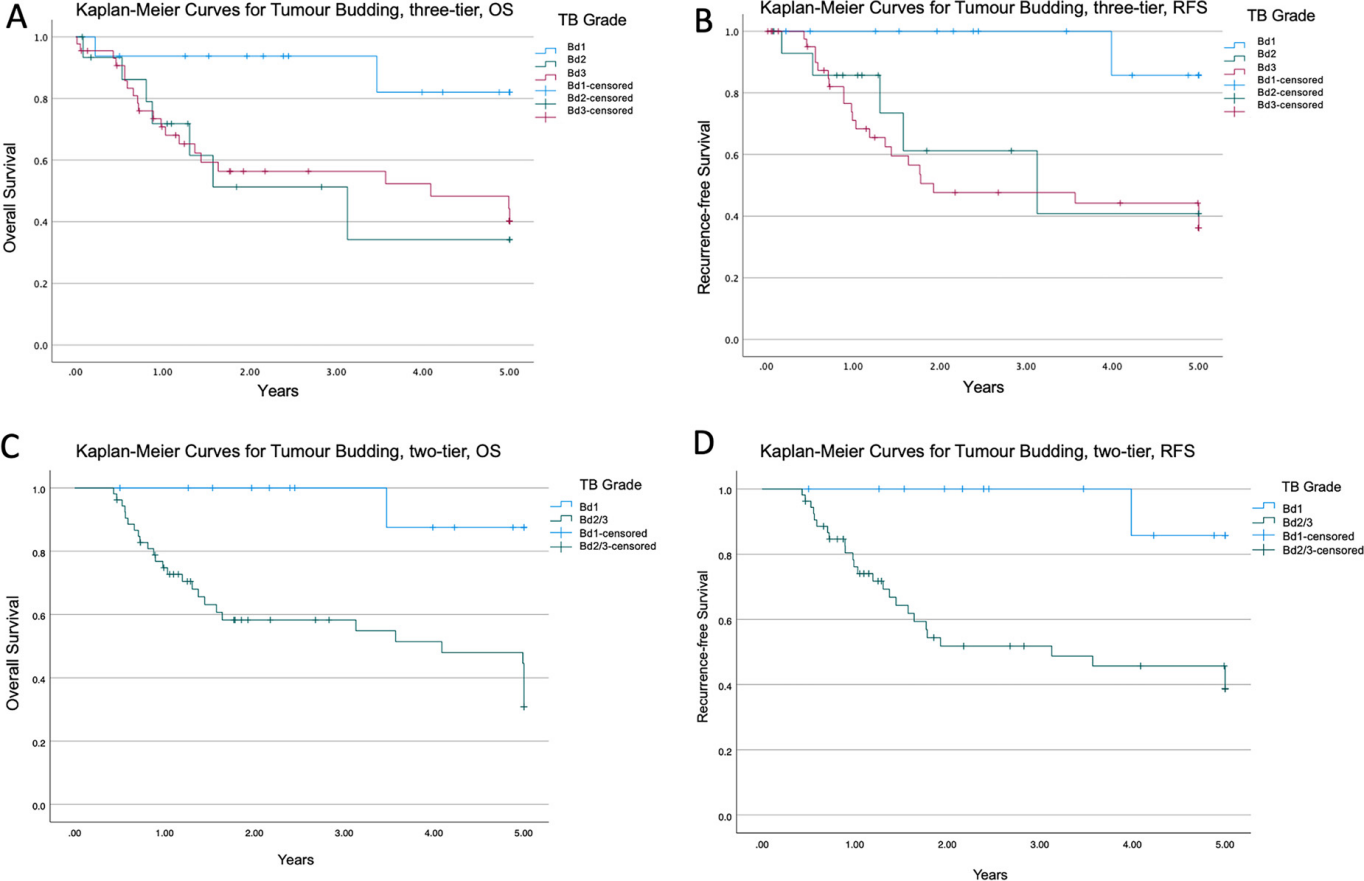
Kaplan-Meier survival analysis for tumour budding tumour budding using three-tier and two-tier grading. A. Overall survival stratified by three-tier grading in intestinal-type gastric adenocarcinoma, *P* = .031. B. Recurrence-free survival stratified by three-tier grading in intestinal-type gastric adenocarcinoma, *P* = .014. C. Overall survival stratified by two-tier grading in intestinal-type gastric adenocarcinoma, *P* = .004. D. Recurrence-free survival stratified by two-tier grading in intestinal-type gastric adenocarcinoma, *P* = .005.

Follow-up data were available for 68 patients from 30 days post-surgery for which survival analysis was performed. Median OS was 20.52 months. The OS outcome was higher in patients with Bd1 compared to Bd2 and Bd3 (Kaplan Meier, log rank test, *P* = .031, Figure 3A). Similarly, Bd1 was associated with a higher RFS compared to Bd2 and Bd3 (Kaplan Meier, log rank test, *P* = .014, Figure 3B). Subgroup analysis of only pure intestinal-type gastric adenocarcinoma, excluding cases with mixed histology (n = 58), showed a trend towards association with OS but did not reach statistical significance (Kaplan Meier, log rank test, *P* = .069), though there was a significant association with RFS (Kaplan Meier, log rank test, *P* = .041). When tumour budding was stratified into low (Bd1) versus high (Bd2/3) by grouping intermediate and high-grade tumour budding, OS and RFS were significantly higher in Bd1 compared to Bd2/3 (Kaplan Meier, log rank test, *P* = .004 and *P* *=* *.*005, respectively, Figure 3C-D). Similar trends were seen in subgroup analysis of pure intestinal-type gastric adenocarcinoma with stratification into low (Bd1) versus grouped high-grade tumour budding (Bd2/3) for OS and RFS (Kaplan Meier, log rank test, *P* = .032 and *P* = .011 respectively).

**Figure 4.**
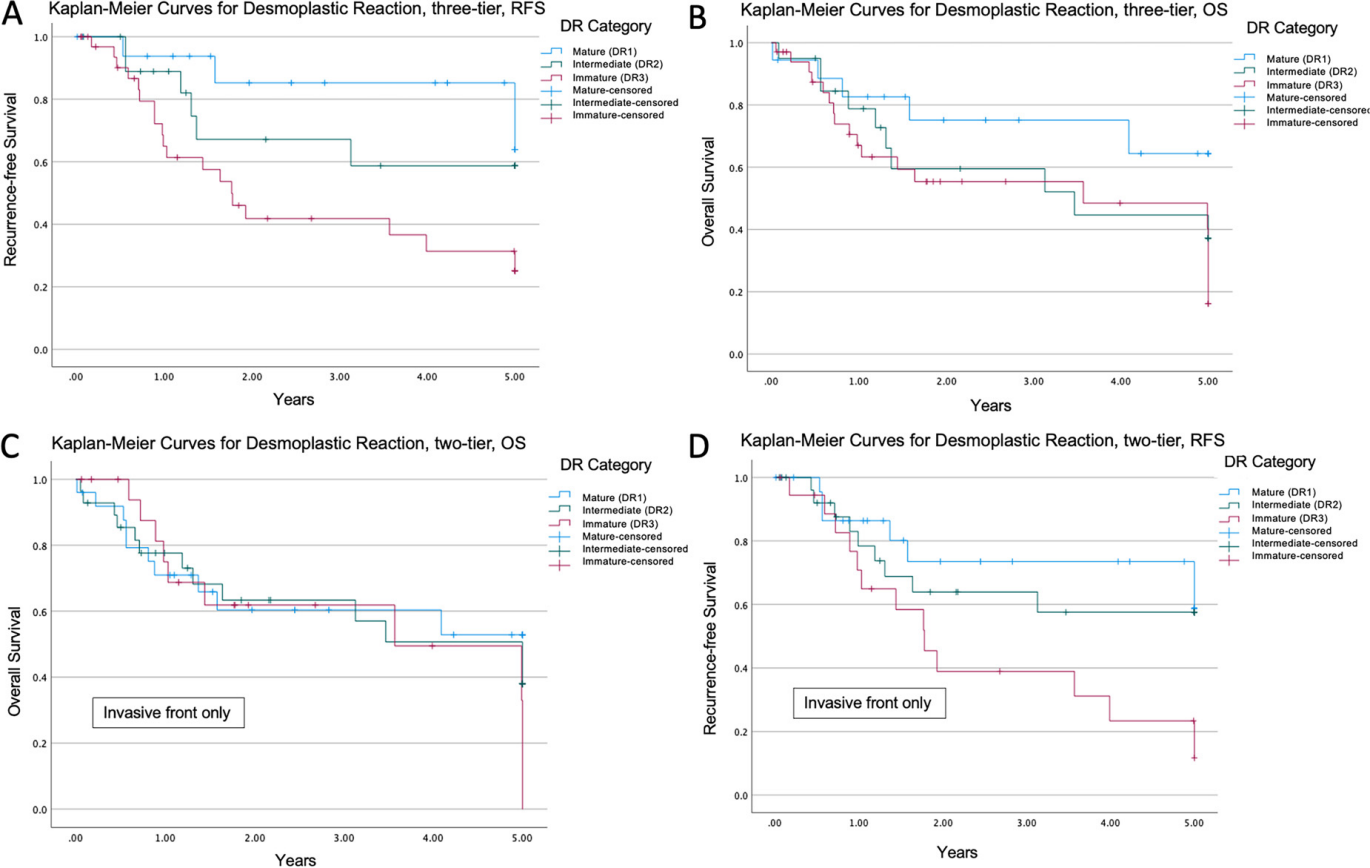
Kaplan-Meier survival analysis for desmoplastic reactions. A. Recurrence-free survival stratified by desmoplastic reaction in intestinal-type gastric adenocarcinoma, *P* = .012. B. Overall survival stratified by desmoplastic reaction in intestinal-type gastric adenocarcinoma, *P* = .173. C. Overall survival stratified by desmoplastic reaction at the invasive front only, *P* = .682. D. Recurrence-free survival stratified by desmoplastic reaction at the invasive front only, *P* = .029.

RFS was higher in patients with mature peritumoral stroma compared to intermediate and immature stroma (Kaplan Meier, log rank test, *P* = .012, Figure 4A). Although a larger proportion of patients with intermediate and immature stroma died, association with OS was not statistically significant (Kaplan Meier, log rank test, *P* = .173, Figure 4B). When desmoplastic reaction was only assessed at the invasive front (n = 45), excluding keloid-like collagen and myxoid areas found within the main tumour mass, desmoplastic reaction was still associated with RFS (*P* = .029) but not with OS (*P* = .682, Figure 4C-D). In multivariate analysis, tumour budding and desmoplastic reaction were not found to be independent prognostic factors of OS or RFS (Table 2).

**Table 2.** Multivariate Analyses for TB and DR in Intestinal-Type Gastric Adenocarcinoma.

	Hazard ratio (95% Confidence Interval)	*P*-value
pT stage		
T1a	Excluded	
T1b	Reference	
T2	1.197 (0.170-8.427)	0.857
T3	1.396 (0.266-7.318)	0.693
T4a	0.909 (0.131-6.322)	0.923
T4b	6.480 (0.0637-65.920)	0.114
pN stage		
N0	Reference	
N1	1.294 (0.342-4.893)	0.704
N2	4.418 (1.165-16.747)	0.029
N3	15.010 (2.689-83.776)	0.002
LVI	0.971 (0.257-3.668)	0.966
PNI	1.999 (0.494-8.081)	0.331
Mixed histology	1.732 (0.507-5.912)	0.381
TB (3-tier)		
1	Reference	
2	6.831 (0.660-70.743)	0.107
3	3.930 (0.340-45.440)	0.273
DR		
Mature	Reference	
Intermediate	0.654 (0.138-3.102)	0.593
Immature	0.404 (0.052-3.130)	0.386

Abbreviations: TB, tumour budding; DR, desmoplastic reaction grade; Bd, Tumour budding grade; LVI, lympho-vascular invasion; PNI, perineural invasion.

In subgroup survival analysis of node-negative patients only (n = 28), tumour budding and desmoplastic reaction were not significantly associated with OS (*P* = .227 and .195, respectively) and RFS (*P* = .206 and .758, respectively). Tumour budding, when stratified by two-tier grading, trended more towards an association with OS and RFS but was not statistically significant (*P* = .195 and .096, respectively). When excluding 4 patients with metastatic disease, tumour budding and desmoplastic reaction remained significantly associated with RFS (*P* = .020 and .025, respectively) but not with OS (*P* = .066 and .378, respectively). Tumour budding, when stratified by two-tier grading in patients without metastatic disease, was significantly associated with OS and RFS (*P* = .021 and .006, respectively). Subgroup analysis for patients without adjuvant chemotherapy showed significance with RFS for tumour budding and DR (P = .021 and .031, respectively) but not for OS. When tumour budding is stratified by a two-grade (high vs. low) system it was significantly associated with both OS and RFS (P = .037 and .009, respectively).

## Discussion

Tumour budding and peritumoral stromal morphology are characteristics of the tumour microenvironment that play a role in cancer progression and are associated with clinical outcomes. tumour budding has been extensively studied in colorectal carcinoma where it is an established independent prognostic factor based on studies from multiple large cohorts. More recently, tumour budding has been shown to demonstrate similar prognostic power for other malignancies including esophageal, pancreatic, lung, and oral cancers.^28^ desmoplastic reaction has also recently been highlighted as a novel independent prognostic factor in stage II colorectal carcinoma (SACURA trial).^12^

Neither tumour budding nor desmoplastic reaction have been extensively studied in gastric adenocarcinoma. While this may be partly due to a lower volume of gastric adenocarcinoma compared to colorectal carcinoma in most Western centres, it is likely also related to a lack of standardized guidelines for assessment of these factors in the case of desmoplastic reaction and the conceptual difficulty of how to handle diffuse-type tumours, almost all of which are Bd3 by definition, in the case of tumour budding. In 1992 Gabbert and colleagues described “tumour cell dissociation” (TCD) in gastric cancer (n = 445), and as an early precursor to the current tumour budding scoring system, categorized cases semi-quantitatively into four tiers including no TCD, few single cells, moderate single cells and predominantly single cells.^17^ Patients with more TCD showed poorer survival outcomes. However, the assessment of TCD was hindered by the lack of objective thresholds for the four categories in this study and especially because most diffuse-type tumours were placed in the predominantly single cell category, which confounded tumour budding with Lauren classification.

Several subsequent studies have categorized tumour budding in gastric cancer into high- and low-grades with varied thresholds ranging from 5 to 10 buds.^19–22^ Three studies examined several hotspots at the invasive front to obtain an average tumour budding count,^19–21^ while only two have assessed tumour budding in gastric adenocarcinoma using the ITBCC guidelines.^26,27^ In our current study, we applied the ITBCC guidelines in assessing tumour budding using the three-tier system and found statistically significant correlation with survival outcomes. However, our Kaplan Meier analyses showed that survival trends were similar between Bd2 and Bd3 groups. When Bd2 and Bd3 were grouped and compared against Bd1, there was an even more robust correlation with survival outcomes. These data suggest that in gastric adenocarcinoma, the distinction between low grade budding (0-4 buds) and intermediate grade budding (5-9 buds) may be a significant threshold prognostically.

Previous studies on tumour budding in gastric adenocarcinoma have included both diffuse and intestinal type histologies. Subsequently, Kemi et al demonstrated that assessing tumour budding in diffuse-type carcinomas (n = 583) does not show significant prognostic value and is therefore not recommended.^22^ Our current study consequently excluded cases that were classified as diffuse-type and mixed cases where there was less than 50% intestinal-type differentiation, while only assessing tumour budding in the intestinal-type component of mixed cases that were included.

Du and colleagues (n = 621) evaluated tumour budding and desmoplastic reaction characteristics in early submucosal cancers.^24^ However, tumour budding was only documented as present or absent. desmoplastic reaction was categorized based on the presence of lymphoplasmacytic, myxoid and hyaline fibrotic stroma at the deepest point of invasion, although the prognostic significance of the stromal characteristics was not thoroughly discussed. A recent meta-analysis by Guo and colleagues examined data from 7 studies, which showed that high-grade tumour budding, regardless of the definition used, was associated with poorer clinical outcomes in intestinal-type gastric adenocarcinoma and with other pathological prognostic factors such as overall tumour stage, lymph node metastasis, and LVI.^25^

There are less data on stromal characteristics in gastric cancer. Kemi and colleagues (n = 583) were the first to demonstrate stromal maturity as an independent prognostic factor in gastric adenocarcinoma using a two-tier system to categorize tumours into mature and immature stroma groups.^23^ In that study, immature stroma was defined as the presence of keloid-like collagen, and it was noted that there were few tumours that contained myxoid stroma. In contrast, we found, using the criteria described by Ueno et al in colorectal carcinoma,^7^ that myxoid stroma was present in 46% of cases. This discrepancy may be due to a difference in the definition of myxoid stroma or, more likely, the minimum amount required to designate the tumour as having immature stroma, as, using the Ueno method, we required only a single full 40x field to be occupied by the most immature stromal type. Hacking and colleagues proposed a modified three-tier method of assessing desmoplastic reaction in colorectal carcinoma by using 0 (no immature desmoplastic stromal reaction), 1 (minimal immature desmoplastic stromal reaction), and 2 (predominant immature desmoplastic stromal reaction).^9,10,29,30^ This system considers the amount of myxoid stroma overall, rather than only the most immature stroma type occupying a 40x field. The authors were able to demonstrate that with this new scoring system, patients could be stratified into risk groups associated with disease-free survival. However, most data on desmoplastic reaction have used the conventional method first described by Ueno and colleagues, as we employed in this current study.^7^ The variation in criteria and occasional discrepancies in predicting prognosis between studies demonstrates the need for a standardized approach to assessing desmoplastic reaction in future studies before it can be considered for use in routine clinical practice.

In the current study, we examined stromal maturity both at the invasive front and within the main tumour mass and used the most immature stromal type in our analysis. Ueno and colleagues examine stromal maturity only at the invasive front.^7^ In our cohort, we found 22 cases where keloid-like collagen or myxoid areas were found within the main tumour mass but not at the invasive front. Our data suggest that assessing tumour at both locations may have a better correlation with recurrence-free survival. Kemi and colleagues have similarly assessed stroma both within the tumour mass and at the invasive front, showing similar associations with and overall survival.^23^ More direct comparative analyses between these two methods should be considered in future studies.

Our study adds to a growing body of literature demonstrating prognostic significance of tumour budding and desmoplastic reaction in gastric adenocarcinoma but also has several limitations. First, the sample size is smaller than other studies reviewed above, largely due to our inclusion criteria which excluded patients who received neoadjuvant therapy, which is being used increasingly commonly in gastric cancer. Eight patients were lost to follow-up, which additionally limited our statistical power due to our small sample size. We did not observe a statistically significant effect on prognosis in multivariate analyses using this limited cohort. However, we were still able to demonstrate sizeable effects for both tumour budding and desmoplastic reaction with a relatively small number of samples. This may be due to the fact that the cases were taken from a single institution, where surgical techniques, adjuvant chemotherapy decisions, and pathologic processing of resected specimens were performed in a consistent and standardized manner. Second, some cohort variables were unknown, including EBV and MSI status, which were unavailable for testing at the time of this study. Although these features are important for molecular subtyping of gastric cancers, they constitute a small proportion of gastric cancers overall and the associated statistical effect on clinical outcomes would likely be very small, if any, in our cohort, where only a few cases in each of these groups would be expected. HER2 status was available for 14 patients, with only four showing positivity and therefore the potential effect of HER2 targeted therapy could not be significantly assessed within our cohort. Third, only one observer performed tumour budding and desmoplastic reaction assessments and therefore we could not assess interobserver variability, although ITBCC consensus recommendations have been shown to have reasonable interobserver agreement in colorectal carcinoma. Assessment of desmoplastic reaction is not as well defined and further studies with larger cohorts and interobserver variability analyses would add to our current knowledge of its reproducibility.

Assessment of tumour budding and desmoplastic reaction are not well studied in patients with neoadjuvant therapy and thus, these patients were excluded from this study. However, many patients with gastric cancer are now treated with neoadjuvant therapy and exploring features of the tumour microenvironment in these patients may be helpful in further stratifying risk of recurrence and mortality.

## Conclusion

Tumour budding and desmoplastic reaction have been shown to be associated with poor prognostic outcomes in colorectal carcinoma and more recently in gastric cancer. In this study of a small but well-controlled cohort of surgically resected intestinal-type gastric adenocarcinoma from a single institution, we provide further support for their prognostic value in gastric adenocarcinoma by showing that tumour budding and desmoplastic reaction were both associated with tumour stage, lymphovascular invasion, and perineural invasion. Tumour budding was associated with OS and RFS, while desmoplastic reaction was associated with RFS in univariate analyses.

## References

[1] Bray F Ferlay J Soerjomataram I Siegel RL Torre LA Jemal A . Global cancer statistics 2018: GLOBOCAN estimates of incidence and mortality worldwide for 36 cancers in 185 countries. CA Cancer J Clin. 2018;68(6):394‐424. 30207593 10.3322/caac.21492

[2] Amin MB Edge S Greene F , et al. AJCC Cancer Staging Manual. 8th ed. Springer; 2017.

[3] Prall F . Tumour budding in colorectal carcinoma. Histopathology. 2007;50(1):151‐162. 17204028 10.1111/j.1365-2559.2006.02551.x

[4] Mitrovic B Schaeffer DF Riddell RH Kirsch R . Tumor budding in colorectal carcinoma: time to take notice. Mod Pathol. 2012;25(10):1315‐1325. 22790014 10.1038/modpathol.2012.94

[5] Lugli A Kirsch R Ajioka Y , et al. Recommendations for reporting tumor budding in colorectal cancer based on the international tumor budding consensus conference (ITBCC) 2016. Mod Pathol. 2017;30:1299‐1311. 28548122 10.1038/modpathol.2017.46

[6] Ueno H Jones A Jass JR Talbot IC . Clinicopathological significance of the ‘keloid-like’ collagen and myxoid stroma in advanced rectal cancer. Histopathology. 2002;40(4):327‐334. 11943016 10.1046/j.1365-2559.2002.01376.x

[7] Ueno H Kanemitsu Y Sekine S , et al. Desmoplastic pattern at the tumor front defines poor-prognosis subtypes of colorectal cancer. Am J Surg Pathol. 2017;41(11):1506‐1512. 28877064 10.1097/PAS.0000000000000946

[8] Ueno H Kanemitsu Y Sekine S , et al. A multicenter study of the prognostic value of desmoplastic reaction categorization in stage II colorectal cancer. Am J Surg Pathol. 2019;43(8):1015‐1022. 31094924 10.1097/PAS.0000000000001272

[9] Hacking SM Ebare K Angert M , et al. Immature stroma and prognostic profiling in colorectal carcinoma: development and validation of novel classification systems. Pathol Res Pract. 2020;216(7):152970. 32534718 10.1016/j.prp.2020.152970

[10] Hacking SM Chakraborty B Nasim R Vitkovski T Thomas R . A holistic appraisal of stromal differentiation in colorectal cancer: biology, histopathology, computation, and genomics. Pathol Res Pract. 2021:153378. 33690050 10.1016/j.prp.2021.153378

[11] González IA Bauer PS Liu J Chatterjee D . Intraepithelial tumour infiltrating lymphocytes are associated with absence of tumour budding and immature/myxoid desmoplastic reaction, and with better recurrence-free survival in stages I–III colorectal cancer. Histopathology. 2021;78(2):252‐264. 32654226 10.1111/his.14211 PMC7775349

[12] Ueno H Ishiguro M Nakatani E , et al. Prognostic value of desmoplastic reaction characterisation in stage II colon cancer: prospective validation in a phase 3 study (SACURA trial). Br J Cancer. 2021;124:1088‐1097. 33414540 10.1038/s41416-020-01222-8 PMC7960987

[13] Kalluri R . EMT: when epithelial cells decide to become mesenchymal-like cells. J Clin Invest. 2009;119(6):1417‐1419. 19487817 10.1172/JCI39675 PMC2689122

[14] Zlobec I Lugli A . Epithelial mesenchymal transition and tumor budding in aggressive colorectal cancer: tumor budding as oncotarget. Oncotarget. 2010;1(7):651. 21317460 10.18632/oncotarget.199 PMC3248128

[15] De Smedt L Palmans S Andel D , et al. Expression profiling of budding cells in colorectal cancer reveals an EMT-like phenotype and molecular subtype switching. Br J Cancer. 2017;116(1):58‐65. 27884016 10.1038/bjc.2016.382 PMC5220148

[16] Li H Xu F Li S Zhong A Meng X Lai M . The tumor microenvironment: an irreplaceable element of tumor budding and epithelial-mesenchymal transition-mediated cancer metastasis. Cell Adh Migr. 2016;10(4):1‐3. 26743180 10.1080/19336918.2015.1129481 PMC4986710

[17] Gabbert HE Meier S Gerharz CD Hommel G . Tumor-cell dissociation at the invasion front: a new prognostic parameter in gastric cancer patients. Int J Cancer. 1992;50(2):202‐207. 1730514 10.1002/ijc.2910500208

[18] Tanaka K Shimura T Kitajima T , et al. Tropomyosin-related receptor kinase B at the invasive front and tumour cell dedifferentiation in gastric cancer. Br J Cancer. 2014;110(12):2923‐2934. 24853179 10.1038/bjc.2014.228 PMC4056051

[19] Gulluoglu M Yegen G Ozluk Y , et al. Tumor budding is independently predictive for lymph node involvement in early gastric cancer. Int J Surg Pathol. 2015;23:349‐358. 25911564 10.1177/1066896915581200

[20] Che K Zhao Y Qu X , et al. Prognostic significance of tumor budding and single cell invasion in gastric adenocarcinoma. Onco Targets Ther. 2017;10:1039‐1047. 28255247 10.2147/OTT.S127762 PMC5325090

[21] Olsen S Jin L Fields RC , et al. Tumor budding in intestinal-type gastric adenocarcinoma is associated with nodal metastasis and recurrence. Hum Pathol. 2017;68:26‐33. 28428104 10.1016/j.humpath.2017.03.021

[22] Kemi N Eskuri M Ikäläinen J Karttunen TJ Kauppila JH . Tumor budding and prognosis in gastric adenocarcinoma. Am J Surg Pathol. 2019;43(2):229‐234. 30334831 10.1097/PAS.0000000000001181

[23] Kemi N Eskuri M Pohjanen VM Karttunen TJ Kauppila JH . Histological assessment of stromal maturity as a prognostic factor in surgically treated gastric adenocarcinoma. Histopathology. 2019;75(6):882‐889. 31173384 10.1111/his.13934

[24] Du M Chen L Cheng Y , et al. Tumor budding and other risk factors of lymph node metastasis in submucosal early gastric carcinoma. Am J Surg Pathol. 2019;43(8):1074‐1082. 31094925 10.1097/PAS.0000000000001276

[25] Guo YX Zhang ZZ Zhao G Zhao EH . Prognostic and pathological impact of tumor budding in gastric cancer: a systematic review and meta-analysis. World J Gastrointest Oncol. 2019;11(10):898. 31662828 10.4251/wjgo.v11.i10.898 PMC6815922

[26] Ulase D Heckl S Behrens HM Krüger S Röcken C . Prognostic significance of tumour budding assessed in gastric carcinoma according to the criteria of the international tumour budding consensus conference. Histopathology. 2020;76(3):433‐446. 31538348 10.1111/his.13997

[27] Dao TV Nguyen CV Nguyen QT , et al. Evaluation of tumor budding in predicting survival for gastric carcinoma patients in Vietnam. Cancer Control. 2020;27(1):1073274820968883. 33136444 10.1177/1073274820968883 PMC7791444

[28] Berg KB Schaeffer DF . Tumor budding as a standardized parameter in gastrointestinal carcinomas: more than just the colon. Mod Pathol. 2018;31(6):862‐872. 29403085 10.1038/s41379-018-0028-4

[29] Son GM Kwon MS Shin DH Shin N Ryu D Kang CD . Comparisons of cancer-associated fibroblasts in the intratumoral stroma and invasive front in colorectal cancer. Medicine (Baltimore). 2019;98(18):e15164. 31045759 10.1097/MD.0000000000015164 PMC6504275

[30] Shin N Son GM Shin DH , et al. Cancer-associated fibroblasts and desmoplastic reactions related to cancer invasiveness in patients with colorectal cancer. Ann Coloproct. 2019;35(1):36. 10.3393/ac.2018.09.10 PMC6425246 30879282

